# Catalysing sustainable fuel and chemical synthesis

**DOI:** 10.1007/s13203-014-0056-z

**Published:** 2014-04-09

**Authors:** Adam F. Lee

**Affiliations:** European Bioenergy Research Institute, Aston University, Aston Triangle, Birmingham, B4 7ET UK

**Keywords:** Heterogeneous catalysis, Biofuels, Biodiesel, Selective oxidation, Alcohols

## Abstract

Concerns over the economics of proven fossil fuel reserves, in concert with government and public acceptance of the anthropogenic origin of rising CO_2_ emissions and associated climate change from such combustible carbon, are driving academic and commercial research into new sustainable routes to fuel and chemicals. The quest for such sustainable resources to meet the demands of a rapidly rising global population represents one of this century’s grand challenges. Here, we discuss catalytic solutions to the clean synthesis of biodiesel, the most readily implemented and low cost, alternative source of transportation fuels, and oxygenated organic molecules for the manufacture of fine and speciality chemicals to meet future societal demands.

## Introduction

Sustainability, in essence the development of methodologies to meet the needs of the present without compromising those of future generations has become a watchword for modern society, with developed and developing nations and multinational corporations promoting international research programmes into sustainable food, energy, materials and even city planning. In the context of energy and materials (specifically synthetic chemicals), despite significant growth in proven and predicted fossil fuel reserves over the next two decades, notably heavy crude oil, tar sands, deepwater wells, and shale oil and gas, there are great uncertainties in the economics of their exploitation via current extraction methodologies, and crucially, an increasing proportion of such carbon resources (estimates vary between 65 and 80 % [1–3]) cannot be burned without breaching the UNFCC targets for a 2 °C increase in mean global temperature relative to the pre-industrial level [4, 5]. There is clearly a tightrope to walk between meeting rising energy demands, predicted to rise 50 % globally by 2040 [6] and the requirement to mitigate current CO_2_ emissions and hence climate change. The quest for sustainable resources to meet the demands of a rapidly rising global population represents one of this century’s grand challenges [7, 8].

**Scheme 1:**
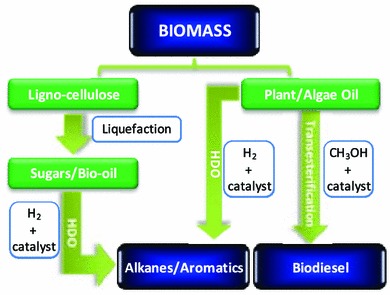
Chemical conversion routes for the co-production of chemicals and transportation fuels from biomass

While many alternative sources of renewable energy have the potential to meet future energy demands for stationary power generation, biomass offers the most readily implemented, low cost solution to a drop-in transportation fuel for blending with/replacing conventional diesel [9] via carbohydrate hydrodeoxygenation (HDO) or lipid transesterification illustrated in Scheme 1. First generation bio-based fuels derived from edible plant materials received much criticism over the attendant competition between land usage for fuel crops versus traditional agricultural cultivation [10]. Deforestation practices, notably in Indonesia, wherein vast tracts of rainforest and peat land are being cleared to support palm oil plantations have also provoked controversy [11]. To be considered sustainable, second generation bio-based fuels and chemicals are sought that use biomass sourced from non-edible components of crops, such as stems, leaves and husks or cellulose from agricultural or forestry waste. Alternative non-food crops such as switchgrass or *Jatropha curcas* [12], which require minimal cultivation and do not compete with traditional arable land or drive deforestation, are other potential candidate biofuel feedstocks. There is also growing interest in extracting bio-oils from aquatic biomass, which can yield 80–180 times the annual volume of oil per hectare than that obtained from plants [13]. Approximately 9 % of transportation energy needs are predicted to be met via liquid bio-fuels by 2030 [14]. While the abundance of land and aquatic biomass, and particularly of agricultural, forestry and industrial waste, is driving the search for technologies to transform lignocellulose into fuels and chemical, energy and atom-efficient processes to isolate lignin and hemicellulose from the more tractable cellulose component, remain to be identified [15]. Thermal pyrolysis offers one avenue by which to obtain transportation fuels, and wherein catalysis will undoubtedly play a significant role in both pyrolysis of raw biomass and subsequent upgrading of bio-oils via deoxygenation and carbon chain growth. Catalytic depolymerisation of lignin may also unlock opportunities for the production of phenolics and related aromatic compounds for fine chemical and pharmaceutical applications [16].

**Scheme 2:**
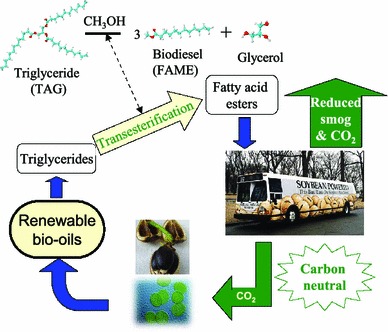
Carbon cycle for biodiesel production from renewable bio-oils via catalytic transesterification

Biodiesel is a clean burning and biodegradable fuel which, when derived from non-food plant or algal oils or animal fats, is viewed as a viable alternative (or additive) to current petroleum-derived diesel [17]. Commercial biodiesel is currently synthesised via liquid base-catalysed transesterification of C_14_–C_20_ triacylglyceride (TAG) components of lipids with C_1_–C_2_ alcohols [18–21] into fatty acid methyl esters (FAMEs) which constitute biodiesel as shown in Scheme 2, alongside glycerol as a potentially valuable by-product [22]. While the use of higher (e.g. C_4_) alcohols is also possible [23], and advantageous in respect of producing a less polar and corrosive FAME [24] with reduced cloud and pour points [25], the current high cost of longer chain alcohols, and difficulties associated with separating the heavier FAME product from unreacted alcohol and glycerol, remain problematic. Unfortunately, homogeneous acid and base catalysts can corrode reactors and engine manifolds, and their removal from the resulting biofuel is particularly problematic and energy intensive, requiring aqueous quench and neutralisation steps which result in the formation of stable emulsions and soaps [9, 26, 27]. Such homogeneous approaches also yield the glycerine by-product, of significant potential value to the pharmaceutical and cosmetic industries, in a dilute aqueous phase contaminated by inorganic salts. Heterogeneous catalysis has a rich history of facilitating energy efficient selective molecular transformations and contributes to 90 % of chemical manufacturing processes and to more than 20 % of all industrial products [28, 29]. While catalysis has long played a pivotal role in petroleum refining and petrochemistry, in a post-petroleum era, it will face new challenges as an enabling technology to overcoming the engineering and scientific barriers to economically feasible routes to bio-fuels. The utility of solid base and acid catalysts for biodiesel production has been extensively reviewed [20, 30–33], wherein they offer improved process efficiency by eliminating the need for quenching steps, allowing continuous operation [34], and enhancing the purity of the glycerol by-product. Technical advances in catalyst and reactor design remain essential to utilise non-food based feedstocks and thereby ensure that biodiesel remains a key player in the renewable energy sector for the 21st century. Select pertinent developments in tailoring the nanostructure of solid acid and base catalysts for TAG transesterification to FAMEs and the related esterification of free fatty acid (FFAs) impurities common in bio-oil feedstocks are therefore discussed herein.

**Scheme 3:**
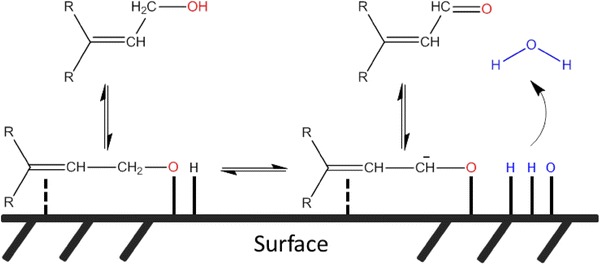
Cartoon depicting the atom-efficient, chemoselective aerobic selective oxidation of allylic alcohols to aldehydes over a heterogeneous catalyst

Biomass also offers the only non-fossil fuel route to organic molecules for the manufacture of bulk, fine and speciality chemicals and polymers [35] required to meet societal demands for advanced materials [8, 36]. The production of such highly functional molecules, whether derived from petroleum feedstocks, requires chemoselective transformations in which e.g. specific heteroatoms or functional groups are incorporated or removed without compromising the underpinning molecular properties. The selective oxidation (selox) of alcohols, carbohydrates and related α,β-unsaturated substrates represent an important class of reactions that underpin the synthesis of valuable chemical intermediates [37, 38]. The scientific, technological and commercial importance of green chemistry presents a significant challenge to traditional selox methods, which previously employed hazardous and toxic stoichiometric oxidants including permanganates, chromates and peroxides, with concomitant poor atom efficiencies and requiring energy-intensive separation steps to obtain the desired carbonyl or acid product. Alternative heterogeneous catalysts utilising oxygen or air as the oxidant offer vastly improved activity, selectivity and overall atom efficiency in alcohol selox (Scheme 3), but are particularly demanding due to the requirement to activate molecular oxygen and C–O bonds in close proximity at a surface in a solid–liquid–gas environment [39–41], and must also be scalable in terms of both catalyst synthesis and implementation. For example, continuous flow microreactors have been implemented in both homogeneous and heterogeneous aerobic selox, providing facile catalyst recovery from feedstreams for the latter [42, 43], but their scale-up/out requires complex manifolding to ensure adequate oxygen dissolution uniform reactant mixing and delivery [44, 45]. Efforts to overcome mass transport and solubility issues inherent to 3-phase catalysed oxidations have centred around the use of supercritical carbon dioxide to facilitate rapid diffusion of substrates to and products from the active catalyst site at modest temperatures [46] affording enhanced turnover frequencies (TOFs), selectivity and on-stream performance versus conventional batch operation in liquid organic solvents [47–51].

The past decade has seen significant progress in understanding the fundamental mode of action of Platinum Group Metal heterogeneous catalysts for aerobic selox and the associated reaction pathways and deactivation processes [41]. This insight has been aided by advances in analytical methodologies, notably the development of in situ or *operando* (under working conditions) spectroscopic [52–54] /microscopic [55–58] tools able to provide quantitative, spatio-temporal information on structure–function relations of solid catalysts in the liquid and vapour phase. Parallel improvements in inorganic synthetic protocols offer finer control over preparative methods to direct the nanostructure (composition, morphology, size, valence and support architecture) of palladium catalysts [59–61] and thereby enhance activity, selectivity and lifetime in an informed manner. Ultimately, heterogeneous catalysts may offer significant advantages over homogeneous analogues in respect of initial catalyst cost, product separation, and metal recovery and recyclability [62]. Catalyst development can thus no longer be considered simply a matter of reaction kinetics, but as a clean technology wherein all aspects of process design, such as solvent selection, batch/flow operation, catalyst recovery and waste production and disposal are balanced [63]. The efficacy of Platinum Group Metals (PGMs) surfaces towards the liquid phase oxidation of alcohols has been known for over 50 years [64], and the development of heterogeneous platinum selox catalysts (and more recently coinage metals such as gold [65, 66]) the subject of recent reviews [39, 67–69] hence only palladium selox catalysis is described herein.

## Heterogeneously catalysed routes to biodiesel

### Solid acid catalysed biodiesel synthesis

A wide range of inorganic and polymeric solid acids are commercially available, however, their application for the transesterification of oils into biodiesel has only been recently explored, in part reflecting their lower activity compared with base-catalysed routes [27], in turn necessitating higher reaction temperatures to deliver suitable conversions. While their activities are generally low, solid acids have the advantage that they are less sensitive to FFA contaminants than their solid base analogues, and hence can operate with unrefined feedstocks containing 3–6 wt% FFAs [27]. In contrast to solid bases which require feedstock pretreatment to remove fatty acid impurities, solid acids are able to esterify FFAs through to FAME in parallel with transesterification major TAG components without soap formation and thus reduce the number of processing steps to biodiesel [70–72].

**Fig. 1:**
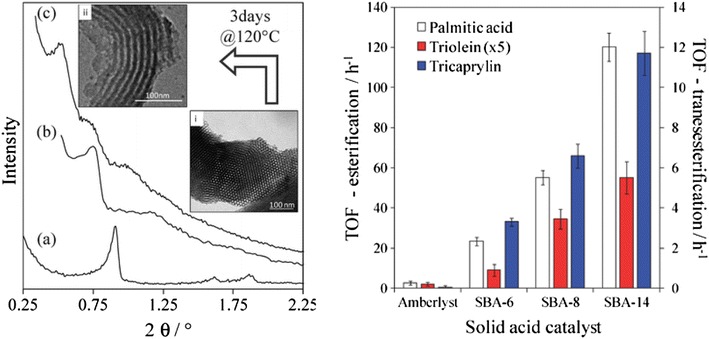
(*Left*) Low angle powder X-ray diffraction patterns and transmission electron micrographs of propylsulfonic acid functionalised SBA-15 silicas as a function of pore diameter; and (*right*) corresponding catalytic activity in FFA esterification and TAG transesterification compared to a commercial solid acid resin. Adapted from reference [80] with permission from The Royal Society of Chemistry

Mesoporous silicas from the SBA family [73] have been examined for biodiesel synthesis, and include materials grafted with sulfonic acid groups [74, 75] or SO_4_/ZrO_2_ surface coatings [76]. Phenyl and propyl sulfonic acid SBA-15 catalysts are particularly attractive materials with activities comparable to Nafion and Amberlyst resins in palmitic acid esterification [77]. Phenylsulfonic acid functionalised silica is reportedly more active than their corresponding propyl analogues, in line with their respective acid strengths but is more difficult to prepare. Unfortunately, conventionally synthesised sulfonic acid functionalised SBA-15 silicas with pore sizes below ~6 nm possess long, isolated parallel channels and suffer correspondingly slow in-pore diffusion and catalytic turnover in FFA esterification. However, poragens such as trimethylbenzene [78] triethylbenzene or triisopropylbenzene [79] can induce swelling of the Pluronic P123 micelles used to produce SBA-15, enabling ordered mesoporous silicas with diameters spanning 5–30 nm, and indeed ultra-large-pores with a BJH pore diameter as much as 34 nm [79]. This methodology was recently applied to prepare a range of large pore SBA-15 materials employing trimethylbenzene as the poragen, resulting in the formation of highly ordered periodic mesostructures with pore diameters of ~6, 8 and 14 nm [80]. These silicas were subsequently functionalised by mercaptopropyl trimethoxysilane (MPTS) and oxidised with H_2_O_2_ to yield expanded PrSO_3_-SBA-15 catalysts which were effective in both palmitic acid esterification with methanol and tricaprylin and triolein transesterification with methanol under mild conditions. For both reactions, turnover frequencies dramatically increased with pore diameter, and all sulfonic acid heterogeneous catalysts significantly outperformed a commercial Amberlyst resin (Fig. 1). These rate enhancements are attributed to superior mass transport of the bulky FFA and triglycerides within the expanded PrSO_3_-SBA-15. Similar observations have been made over Poly(styrenesulfonic acid)-functionalised ultra-large pore SBA-15 in the esterification of oleic acid with butanol [81].

**Fig. 2:**
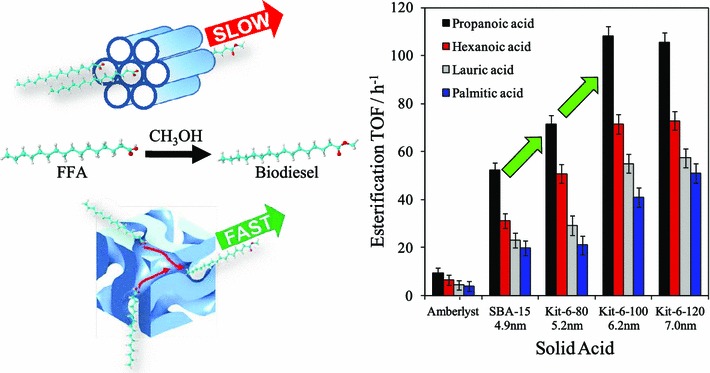
Superior performance of interconnected, mesoporous propylsulfonic acid KIT-6 catalysts for biodiesel synthesis via FFA esterification with methanol versus non-interconnected mesoporous SBA-15 analogue. Adapted from reference [82]. Copyright 2012 American Chemical Society

Improving pore interconnectivity, for example through swapping the *p6* *mm* architecture of SBA-15 for the *Ia3d* of KIT-6 was subsequently explored as an alternative means to enhance in-pore active site accessibility (Scheme 1) for FFA esterification [82]. KIT-6 mesoporous materials exhibit improved characteristics for biomolecule immobilisation [83] reflecting superior diffusion within the interconnected cubic structure. A family of pore-expanded propylsulfonic acid KIT-6 analogues were prepared via MPTS grafting and oxidation and screened for FFA esterification with methanol as a function of alkyl chain length under mild conditions. As-synthesised PrSO_3_H-KIT-6 exhibited respective 40 and 70 % TOF enhancements toward propanoic and hexanoic acid esterification compared with a PrSO_3_H-SBA-15 analogue of comparable (5 nm) pore diameter as a consequence of the improved mesopore interconnectivity. However, pore accessibility remained rate-limiting for esterification of the longer chain lauric and palmitic acids. Hydrothermal aging protocols facilitated expansion of the KIT-6 mesopore up to 7 nm, with consequent doubling of TOFs for lauric and palmitic acid esterification versus PrSO_3_H-SBA-15 (Fig. 2).

**Scheme 4:**
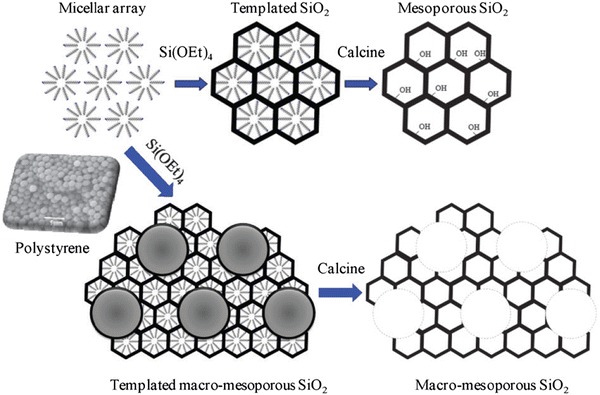
Liquid crystal and polystyrene nanosphere dual surfactant/physical templating route to hierarchical macroporous–mesoporous silicas

**Fig. 3:**
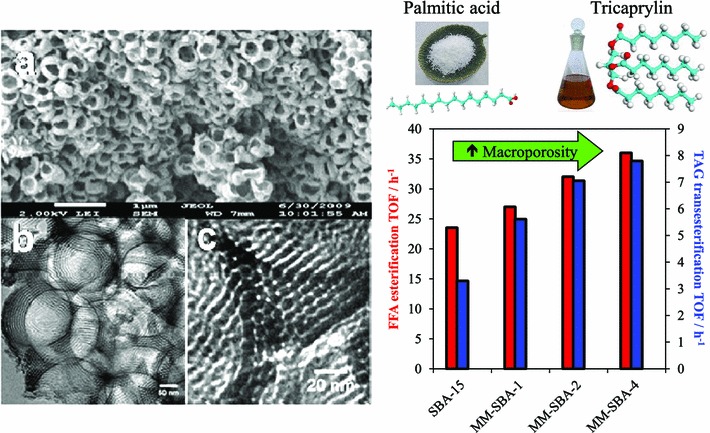
(*Left*) SEM (**a**) and low and high magnification TEM (**b**, **c**) micrographs of a hierarchical macro–mesoporous Pr-SO_3_H-SBA-15; (*right*) corresponding catalytic performance in palmitic acid esterification and tricaprylin transesterification with methanol as a function of macropore density versus a purely mesoporous Pr-SO_3_H-SBA-15. Adapted from reference [93] with permission from The Royal Society of Chemistry

While numerous solid acids have been applied for biodiesel synthesis [27, 32, 84], most materials exhibit micro- and/or mesoporosity which, as illustrated above, are not optimal for accommodating bulky C_16_–C_18_ TAGs of FFAs. For example, incorporation of a secondary mesoporosity into a microporous H-β-zeolite to create a hierarchical solid acid significantly increased catalytic activity by lowering diffusion barriers [85]. Templated mesporous materials are widely used as catalyst supports [86, 87], with SBA-15 silicas popular candidates for reactions pertinent to biodiesel synthesis as previously discussed [75, 77, 88]. However, such surfactant-templated supports possessing long, isolated parallel and narrow channels are ill-suited to efficient in-pore diffusion of bio-oil feedstocks affording poor catalytic turnover. Further improvements in pore architecture are hence required to optimise mass transport of heavier bulky TAGs and FFAs commonly found in plant and algal oils. Simulations demonstrate that in the Knudsen diffusion regime [89], where reactants/products are able to diffuse enter/exit mesopores but experience moderate diffusion limitations, hierarchical pore structures may significantly improve catalyst activity. Materials with interpenetrating, bimodal meso–macropore networks have been prepared using microemulsion [90] or co-surfactant [91] templating routes and are particularly attractive for liquid phase, flow reactors wherein rapid pore diffusion is required. Liquid crystalline (soft) and colloidal polystyrene nanospheres (hard) templating methods have been combined to create highly organised, macro–mesoporous aluminas [92] and ‘SBA-15 like’ silicas [93] (Scheme 4), in which both macro- and mesopore diameters can be independently tuned over the range 200–500 and 5–20 nm, respectively. The resulting hierarchical pore network of a propylsulfonic acid functionalised macro–mesoporous SBA-15 is shown in Fig. 3, wherein macropore incorporation confers a striking enhancement in the rates of tricaprylin transesterification and palmitic acid esterification with methanol, attributed to the macropores acting as transport conduits for reactants to rapidly access PrSO_3_H active sites located within the mesopores.

**Scheme 5:**
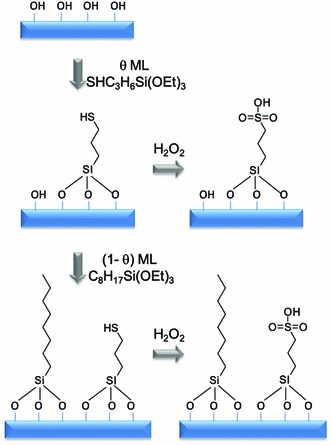
Protocol for the synthesis of sulfonic acid and octyl co-functionalised sulfonic acid MCM-41catalysts. Adapted from reference [101] with permission from The Royal Society of Chemistry

The hydrophilic nature of polar silica surfaces hinders their application for reactions involving apolar organic molecules. This is problematic for TAG transesterification (or FFA esterification) due to preferential in-pore diffusion and adsorption of alcohol versus fatty acid components. Surface hydroxyl groups also favour H_2_O adsorption, which if formed during FFA esterification can favour the reverse hydrolysis reaction and consequent low FAME yields. Surface modification via the incorporation of organic functionality into polar oxide surfaces, or dehydroxylation, can lower their polarity and thereby increase initial rates of acid catalysed transformations of liquid phase organic molecules [94]. Surface polarity can also be tuned by incorporating alkyl/aromatic groups directly into the silica framework, for example polysilsesquioxanes can be prepared via the co-condensation of 1,4-bis(triethoxysilyl)benzene (BTEB), or 1,2-bis(trimethoxysilyl)-ethane (BTME), with TEOS and MPTS in the sol–gel process [95, 96] which enhances small molecule esterification [97] and etherification [98]. The incorporation of organic spectator groups (e.g. phenyl, methyl or propyl) during the sol–gel syntheses of SBA-15 [99] and MCM-41 [100] sulphonic acid silicas is achievable via co-grafting or simple addition of the respective alkyl or aryltrimethoxysilane during co-condensation protocols. An experimental and computational study of sulphonic acid functionalised MCM-41 materials was undertaken to evaluate the effect of acid site density and surface hydrophobicity on catalyst acidity and associated performance [101]. MCM-41 was an excellent candidate due to the availability of accurate models for the pore structure from kinetic Monte Carlo simulations [102], and was modified with surface groups to enable dynamic simulation of sulphonic acid and octyl groups co-attached within the MCM-41 pores. In parallel experiments, two catalyst series were investigated towards acetic acid esterification with butanol (Scheme 5). In one series, the propylsulphonic acid coverage was varied between *θ* (RSO_3_H) = 0–100 % ML over the bare silica (MCM–SO_3_H). For the second octyl co-grafted series, both sulfonic acid and octyl coverages were tuned (MCM–Oc–SO_3_H). These materials allow the effect of lateral interactions between acid head groups and the role of hydrophobic octyl modifiers upon acid strength and activity to be separately probed.

**Fig. 4:**
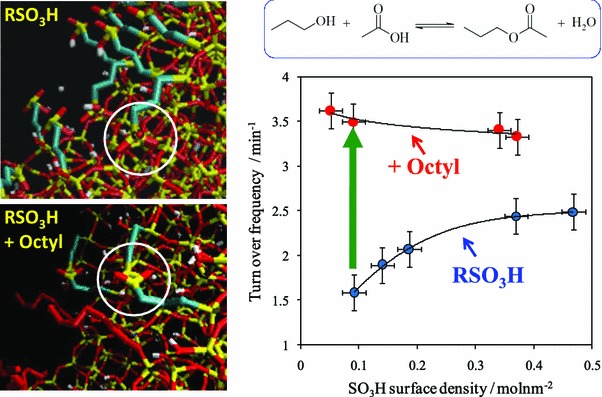
(*Left*) Molecular dynamics simulations of MCM–SO_3_H and MCM–Oc–SO_3_H pore models highlighting the interaction between surface sulfonic acid and hydroxyl groups in the absence of co-grafted octyl chains; (*right*) influence of PrSO_3_H surface density and co-grafted octyl groups on catalytic performance in acetic acid esterification with butanol. Adapted from reference [101] with permission from The Royal Society of Chemistry

To avoid diffusion limitations, butanol esterification with acetic acid was selected as a model reaction (Fig. 4). Ammonia calorimetry revealed that the acid strength of polar MCM–SO_3_H materials increases from 87 to 118 kJ mol^−1^ with sulphonic acid loading. Co-grafted octyl groups dramatically enhance the acid strength of MCM–Oc–SO_3_H for submonolayer SO_3_H coverages, with _ΔH_ads_(NH_3_) rising to 103 kJ mol^−1^. The per site activity of the MCM–SO_3_H series in butanol esterification with acetic acid mirrors their acidity, increasing with SO_3_H content. Octyl surface functionalisation promotes esterification for all MCM–Oc–SO_3_H catalysts, doubling the turnover frequency of the lowest loading SO_3_H material. Molecular dynamic simulations indicate that the interaction of isolated sulphonic acid moieties with surface silanol groups is the primary cause of the lower acidity and activity of submonolayer samples within the MCM–SO_3_H series. Lateral interactions with octyl groups help to re-orient sulphonic acid headgroups into the pore interior, thereby enhancing acid strength and associated esterification activity.

**Fig. 5:**
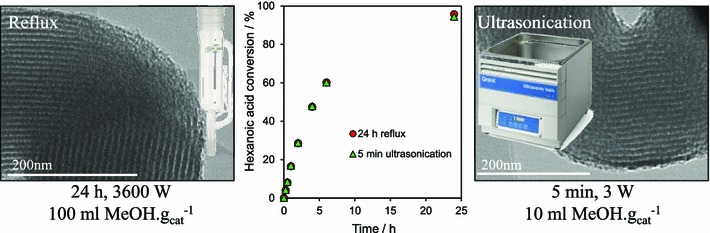
Surfactant template extraction via energy/atom-efficient ultrasonication delivers a one-pot PrSO_3_H-SBA-15 solid acid catalyst with identical structure and reactivity to that obtained by conventional, inefficient reflux. Adapted from reference [103] with permission from The Royal Society of Chemistry

In summary, recent developments in tailoring the structure and surface functionality of sulfonic acid silicas have led to a new generation of tunable solid acid catalysts well-suited to the esterification of short and long chain FFAs, and transesterification of diverse TAGs, with methanol under mild reaction conditions. A remaining challenge is to extend the dimensions and types of pore-interconnectivities present within the host silica frameworks, and to find alternative low cost soft and hard templates to facilitate synthetic scale-up of these catalysts for multi-kg production. Surfactant template extraction is typically achieved via energy-intensive solvent reflux, which results in significant volumes of contaminated waste and long processing times, while colloidal templates often require high temperature calcination which prevents template recovery/re-use and releases carbon dioxide. Preliminary steps towards the former have been recently taken, employing room temperature ultrasonication in a small solvent volume to deliver effective extraction of the P123 Pluronic surfactant used in the preparation of SBA-15 in only 5 min, with a 99.9 % energy saving and 90 % solvent reduction over reflux methods, and without compromising textural, acidic or catalytic properties of the resultant Pr-SO_3_H-SBA-15 in hexanoic acid esterification (Fig. 5) [103].

### Solid base-catalysed biodiesel synthesis

Base catalysts are generally more active than acids in transesterification, and hence are particularly suitable for high purity oils with low FFA content. Biodiesel synthesis using a solid base catalyst in continuous flow, packed bed arrangement would facilitate both catalyst separation and co-production of high purity glycerol, thereby reducing production costs and enabling catalyst re-use. Diverse solid base catalysts are known, notably alkali or alkaline earth oxides, supported alkali metals, basic zeolites and clays such as hydrotalcites and immobilised organic bases [104]. Basicity in alkaline earth oxides is believed to arise from M^2+^–O^2−^ ion pairs present in different coordination environments [105]. The strongest base sites occur at low coordination defect, corner and edge sites, or on high Miller index surfaces. Such classic heterogeneous base catalysts have been extensively tested for TAG transesterification [106] and there are numerous reports on commercial and microcrystalline CaO applied to rapeseed, sunflower or vegetable oil transesterification with methanol [107, 108]. Promising results have been obtained, with 97 % oil conversion achieved at 75 °C [108], however, concern remains over Ca^2+^ leaching under reaction conditions and associated homogeneous catalytic contributions [109], a common problem encountered in metal catalysed biodiesel production which hampers commercialisation [110].

**Fig. 6:**
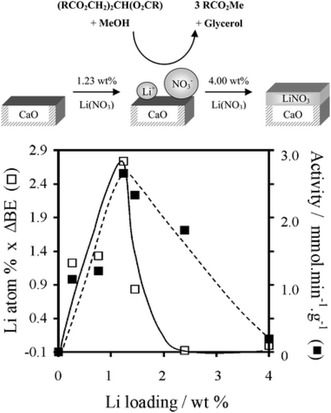
Correlation between evolving surface composition, density of electronically perturbed Li^+^ sites, and corresponding activity in tributyrin transesterification with methanol over Li-doped CaO as a function of Li loading. Adapted from reference [113] with permission from The Royal Society of Chemistry

Alkali-doped CaO and MgO have also been investigated for TAG transesterification [111–113], with their enhanced basicity attributed to the genesis of O^−^ centres following the replacement of M^+^ for M^2+^ and associated charge imbalance and concomitant defect generation. Optimum activity for Li-doped CaO occurs when a saturated Li^+^ monolayer is formed (Fig. 6) [113], although leaching of the alkali promoter remains problematic [114].

**Fig. 7:**
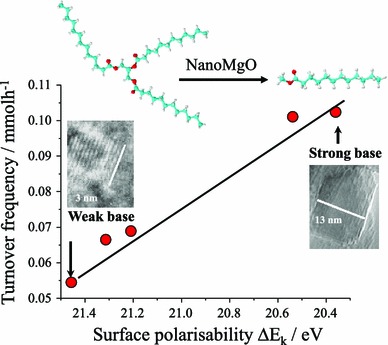
Relationship between surface polarisability of MgO nanocrystals and their turnover frequency towards tributyrin transesterifcation. Adapted from reference [117] with permission from The Royal Society of Chemistry

It is widely accepted that the catalytic activity of alkaline earth oxide catalysts is very sensitive to their preparation, and corresponding surface morphology and/or defect density. For example, Parvulescu and Richards demonstrated the impact of the different MgO crystal facets upon the transesterification of sunflower oil by comparing nanoparticles [115] versus (111) terminated nanosheets [116]. Chemical titration reveals that both morphologies possess two types of base sites, with the nanosheets exhibiting well-defined, medium-strong basicity consistent with their uniform exposed facets and which confer higher FAMe yields during sunflower oil transesterification. Subsequent synthesis, screening and spectroscopic characterisation of a family of size-/shape-controlled MgO nanoparticles prepared via a hydrothermal synthesis revealed small (<8 nm) particles terminate in high coordination (100) facets, and exhibit both weak polarisability and poor activity in tributyrin transesterification with methanol [117]. Calcination drives restructuring and sintering to expose lower coordination stepped (111) and (110) surface planes, which are more polarisable and exhibit much higher transesterification activities under mild conditions. A direct correlation was therefore observed between the surface electronic structure and associated catalytic activity, revealing a pronounced structural preference for (110) and (111) facets (Fig. 7).

**Fig. 8:**
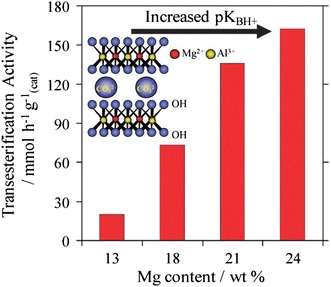
Impact of Mg:Al hydrotalcite surface basicity on their activity towards tributyrin transesterification. Adapted from reference [118] with permission from Elsevier

Hydrotalcites are another class of solid base catalysts that have attracted recent attention because of their high activity and robustness in the presence of water and FFA [118, 119]. Hydrotalcites ([M(II)_1 − *x*_M(III)_*x*_(OH)_2_]^*x*+^(A^*n*^_*x/n*_^−^) mH_2_O) adopt a layered double hydroxide structure with brucite-like Mg(OH)_2_) hydroxide sheets containing octahedrally coordinated M^2+^ and M^3+^ cations and A^*n*−^ anions between layers to balance the overall charge [120], and are conventionally synthesised via co-precipitation from their nitrates using alkalis as both pH regulators and a carbonate source. Mg–Al hydrotalcites have been applied for TAG transesterification of poor and high quality oil feeds [121] such as refined and acidic cottonseed oil (9.5 wt% FFA), and animal fat feed (45 wt% water), delivering 99 % conversion within 3 h at 200 °C. It is important to note that many catalytic studies employing hydrotalcites for transesterification are suspect due to their use of Na or K hydroxide/carbonate solutions to precipitate the hydrotalcite phase. Complete removal of alkali residues from the resulting hydrotalcites is inherently difficult, resulting in parallel ill-defined homogeneous contributions to catalysis arising from leached Na or K [122, 123]. This problem has been overcome by the development of alkali-free precipitation routes using NH_3_OH and NH_3_CO_3_, offering well-defined thermally activated and rehydrated Mg–Al hydrotalcites with compositions spanning *x* = 0.25 − 0.55 [118]. Spectroscopic measurements reveal that increasing the Mg:Al ratio enables the surface charge and accompanying base strength to be systematically enhanced, with a concomitant increase in the rate of tributyrin transesterification under mild reaction conditions (Fig. 8).

**Fig. 9:**
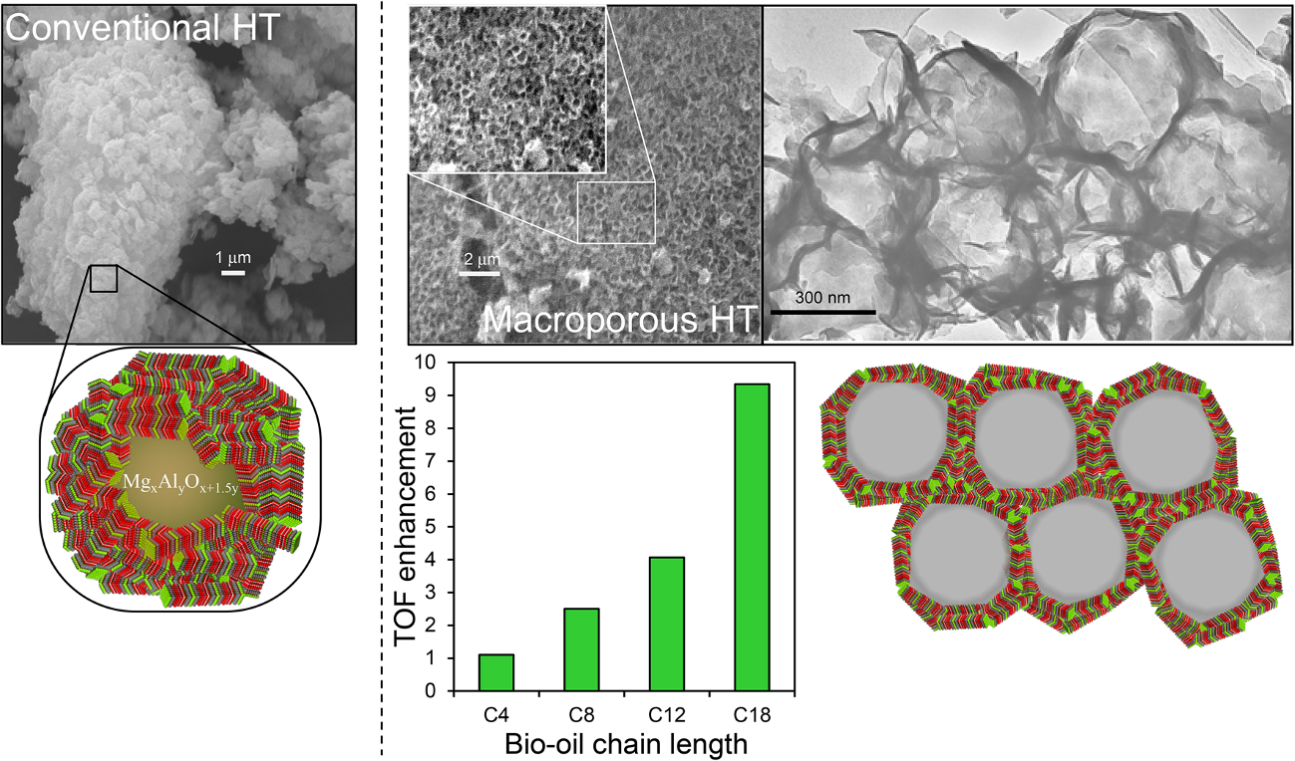
Superior catalytic performance of a hierarchical macroporous–microporous Mg–Al hydrotalcite solid base catalyst for TAG transesterification to biodiesel versus a conventional microporous analogue. Adapted from reference [124] with permission from The Royal Society of Chemistry

In spite of their promise for biodiesel production, conventionally prepared hydrotalcites are microporous, and hence poorly suited to application in the transesterification of bulky C_16_–C_18_ TAG components of bio-oils. This problem was recently tackled by adopting the same hard templating method utilising polystyrene nanospheres described in Scheme 4 to incorporate macroporosity, and thus create a hierarchical macroporous–microporous hydrotalcite solid base catalyst [124]. The introduction of macropores as ‘superhighways’ to rapidly transport heavy TAG oil components to active base sites present at (high aspect ratio) hydrotalcite nanocrystallites, dramatically enhanced turnover frequencies for triolein transesterification compared with that achievable over an analogous Mg–Al microporous hydrotalcite (Fig. 9), reflecting superior mass transport through the hierarchical catalyst.

**Fig. 10:**
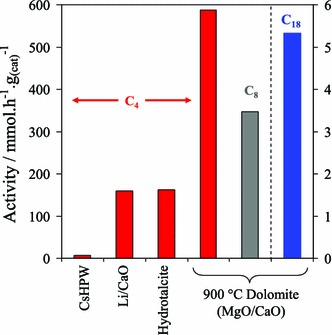
Catalytic activity of calcined Dolomite for the transesterification of short and long chain TAGs with methanol benchmarked against literature solid acid and base catalysts. Reproduced from reference [127] with permission from The Royal Society of Chemistry

In terms of sustainability, it is important to find low cost routes to the synthesis of solid base catalysts that employ earth abundant elements. Dolomitic rock, comprising alternating Mg(CO_3_)–Ca(CO_3_) layers, is structurally very similar to calcite (CaCO_3_), with a high natural abundance and low toxicity, and in the UK is sourced from quarries working Permian dolomites in Durham, South Yorkshire and Derbyshire [125]. In addition to uses in agriculture and construction, dolomite finds industrial applications in iron and steel production, glass manufacturing and as fillers in plastics, paints, rubbers, adhesives and sealants. Catalytic applications for powdered, dolomitic rock offer the potential to further valorise this readily available waste mineral, and indeed dolomite has shown promise in biomass gasification [126] as a cheap, disposable and naturally occurring material that significantly reduces the tar content of gaseous products from gasifiers. Dolomite has also been investigated as a solid base catalyst in biodiesel synthesis [127], wherein fresh dolomitic rock comprised approximately 77 % dolomite and 23 % magnesian calcite. High temperature calcination induced Mg surface segregation, resulting in MgO nanocrystals dispersed over CaO/(OH)_2_ particles, while the attendant loss of CO_2_ increases both the surface area and basicity. The resulting calcined dolomite proved an effective catalyst for the transesterification of C_4_, C_8_ and TAGs with methanol and longer chain C_16–18_ components present within olive oil, with TOFs for tributyrin conversion to methyl butanoate the highest reported for any solid base (Fig. 10). The slower transesterification rates for bulkier TAGs were attributed to diffusion limitations in their access to base sites. Calcined dolomite has also shown promise in the transesterification of canola oil with methanol, achieving 92 % FAME after 3 h reaction with 3 wt% catalyst [128].

In summary, a host of inorganic solid base catalysts have been developed for the low temperature transesterification of triglyceride components of bio-oil feedstocks, offering activities far superior to those achieved via alternative solid acid catalysts to date. However, leaching of alkali and alkaline earth elements and associated catalyst recycling remains a challenge, while improved resilience to water and fatty acid impurities in plant, algal and waste oils feedstocks is required to eliminate additional esterification pre-treatments. To date, only a handful of biodiesel production processes employing heterogeneous catalysts have been commercialised, notably the Esterfip-H process developed by Axens and IFP which utilises a mixture of ZnO and alumina and is operated on a 200 kton per annum scale with parallel production of high quality glycerine [129].

## Palladium catalysed aerobic alcohol selox

### Particle size effects

Within nanocatalysis, the particle size is a well-documented key parameter influencing both activity and selectivity. This reflects the combination of quantum and geometric effects associated with the respective evolution of electronic properties from atomic like to delocalised bands, and shifting population of low to high coordination surface atoms, with increasing nanoparticle size and dimensionality. Kaneda et al. [130] hypothesised that the unique reactivity of 2060 atom Pd clusters supported on titania towards aromatic alcohol selox arose from a distribution of Pd^0^, Pd^+^ and Pd^2+^ surfaces sites, with *π*-bonding interactions between the phenyl group and Pd^2+^ species facilitating subsequent oxidative addition of the O–H bond by neighbouring Pd^0^ and eventual β–hydride elimination. Surface hydride was hypothesised to react with oxygen from a neighbouring Pd_2_O centre forming H_2_O and regenerating the metal site. Optimal activity for cinnamyl alcohol selox to cinnamaldehyde coincided with clusters possessing the maximum fraction of Pd^+^ character.

Particle size dependency was also reported for the catalytic transformation of benzyl alcohol over Pd nanoparticles dispersed on alumina, SiO_2_ and NaX zeolite supports [131, 132]. For Pd/NaX and Pd/SiO_2_-Al_2_O_3_, benzyl alcohol selox was fastest over particles between 3 and 5 nm, whereas geraniol and 2-octanol were structure-insensitive. Systematic studies of particle size effects in cinnamyl and crotyl alcohol selox over amorphous and mesostructured alumina and silica supports have likewise uncovered pronounced size effects in both initial selox rates and TOFs [133–136], which increase monotonically with shrinking nanoparticle diameters (even down to single atoms) [137]. HAADF–STEM analysis reveals atomically dispersed palladium exhibits maximal rates towards benzyl, cinnamyl and crotyl alcohols, with selectivities to their corresponding aldehydes >70 %. The origin of such size effects is revisited below. The use of colloidal Pd nanoclusters for aqueous phase alcohol selox is limited [138–140], wherein Pd aggregation and Pd black formation hinders catalytic performance. However, the successful stabilisation of 3.6 nm Pd nanoclusters is reported using an amphiphilic nonionic triblock copolymer, Pluronic P123; in the selective oxidation of benzyl alcohol, 100 % aldehyde selectivity and high selox rates are achievable, with high catalytic activity maintained with negligible sintering after 13 recycling reactions [141].

### Surface reaction mechanism

**Fig. 11:**
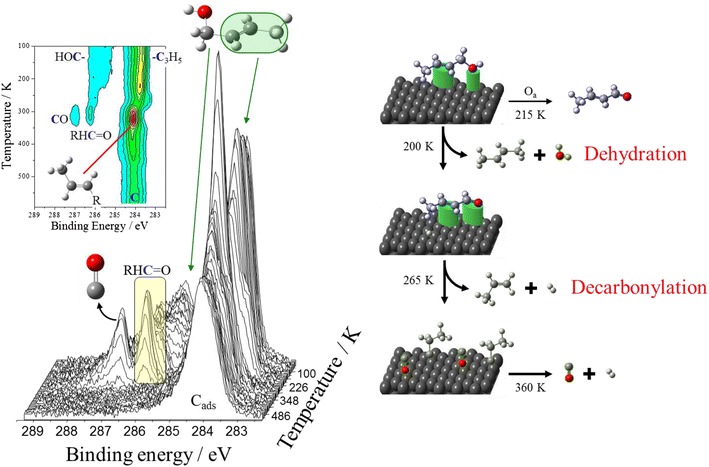
Temperature-programmed C 1s XP spectra of a reacting crotyl alcohol adlayer over Pd(111) highlighting the primary dehydrogenation pathway and competing decarbonylation pathways. Adapted from reference [143]. Copyright 2007 American Chemical Society

The rational design and optimisation of palladium selox catalysts require a microscopic understanding of the active catalytic species responsible for alcohol and oxygen activation, and the associated reaction pathway to the aldehyde/ketone products and any competing processes. A key characteristic of palladium is its ability to perform selox chemistry at temperatures between 60 and 160 °C and with ambient oxygen pressure [39, 142] via the wi dely accepted oxidative dehydrogenation route illustrated in Scheme 3 [39, 67]. Whether O–H or C–H scission of the α-carbon is the first chemical step remains a matter of debate, since the only fundamental studies over well-defined Pd(111) surfaces to date employed temperature-programmed XPS [143] and metastable de-excitation spectroscopy (MDS) [144] with temporal resolutions on the second → minute timescale, over which loss of both hydrogens appears coincident. However, temperature-programmed mass spectrometric [145] and vibrational [146] studies of unsaturated C_1_–C_3_ alcohols implicate O–H cleavage and attendant alkoxy formation over Pd single crystal surfaces as the first reaction step [142, 147]. It is generally held that the resultant hydrogen adatoms react with dissociatively absorbed oxygen to form water, which immediately desorbs at ambient temperature thereby shifting the equilibrium to carbonyl formation [39, 67]. Temperature-programmed XPS studies of crotyl alcohol adsorbed over clean Pd(111) [143] prove that oxidative dehydrogenation to crotonaldehyde occurs at temperatures as low as −60 °C (Fig. 11), with alcohol dehydration to butane only a minor pathway. These ultra-high vacuum measurements also revealed that reactively formed crotonaldehyde undergoes a competing decarbonylation reaction over metallic palladium above 0 °C yielding strongly bound CO and propylidene which may act as site-blockers poisoning subsequent catalytic selox cycles, coincident with evolution of propene into the gas phase. Unexpectedly, pre-adsorbed atomic oxygen switched-off undesired decarbonylation chemistry, promoting facile crotonaldehyde desorption.

### Nature of the active site

**Fig. 12:**
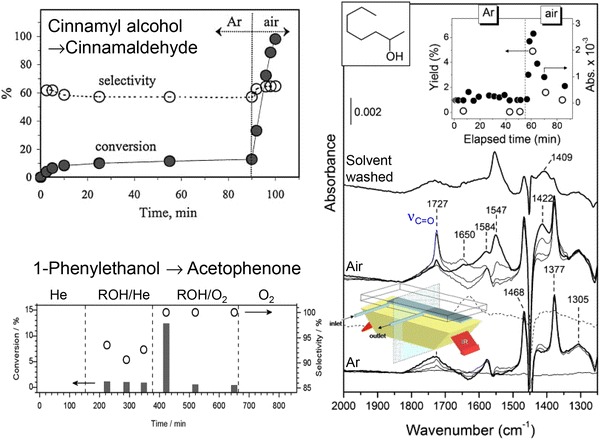
Impact of oxygen on the selective oxidation of (*top left*) cinnamyl alcohol; (*bottom left*) 1-phenylethanol; and (*right*) 2-octanol. Adapted from references [148, 151, 154] with permission from Elsevier

The preceding observation that surface oxygen is not only critical for the removal of hydrogen adatoms but also to suppress decarbonylation of selox products over metallic palladium is in excellent agreement with an in situ ATR-IR study of cinnamyl alcohol selox over Pd/Al_2_O_3_ [148]. In related earlier investigations employing aqueous electrochemical protocols, the same researchers postulated that oxidative dehydrogenation of alcohols requires PGM catalysts in a reduced state, hypothesising that ‘over-oxidation’ was responsible for deactivation of palladium selox catalysts [69]. A subsequent operando X-ray absorption spectroscopy (XAS) study by Grunwaldt et al. [150], bearing remarkable similarity to an earlier study to the author of this review [149], evidenced in situ reduction of oxidised palladium in an as-prepared Pd/Al_2_O_3_ catalyst during cinnamyl alcohol oxidation within a continuous flow fixed-bed reactor. Unfortunately the reaction kinetics were not measured in parallel to explore the impact of palladium reduction, however, a follow-up study of 1-phenylethanol selox employing the same reactor configuration (and oxygen-deficient conditions) evidenced a strong interplay between selox conversion/selectivity and palladium oxidation state [151]. It was concluded that metallic Pd was the catalytically active species, an assertion re-affirmed in subsequent in situ ATR-IR/XAS measurements of benzyl [152–154] and cinnamyl alcohol [155] selox in toluene and under supercritical CO_2_, respectively, wherein the C=O stretching intensity was assumed to track alcohol conversion. It is interesting to note that the introduction of oxygen to the reactant feed in these infrared studies dramatically improved alcohol conversion/aldehyde production (Fig. 12), which was attributed to hydrogen abstraction from the catalyst surface [156, 157] rather than to a change in palladium oxidation state. In contrast to their liquid phase experiments, high pressure XANES and EXAFS measurements of Pd/Al_2_O_3_ catalysed benzyl alcohol selox under supercritical CO_2_ led Grunwaldt and Baiker to conclude that maximum activity arose from particles mainly oxidised in the surface/shelfedge [48].

**Fig. 13:**
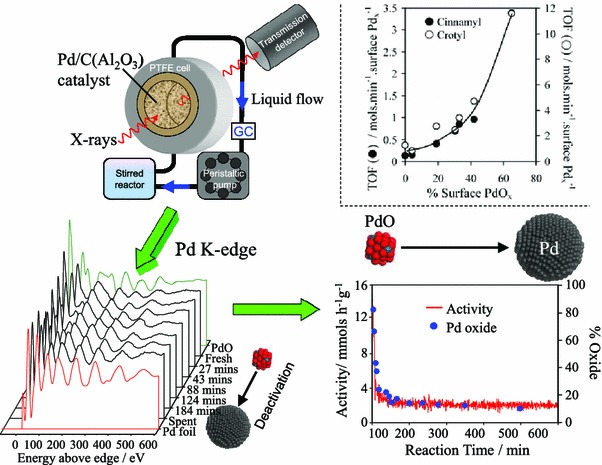
(*Top right*) Dependence of allylic alcohol selox rate upon surface PdO; (*top left*) schematic of operando liquid phase reactor; (*bottom left*) evolution of Pd K-edge XAS of Pd/Al_2_O_3_ catalyst during cinnamyl alcohol aerobic selox; (*bottom right*) temporal correspondence between Pd oxidation state and selox activity in cinnamyl alcohol selox. Adapted from references [133, 134] with permission from The Royal Society of Chemistry

In a parallel research programme, the author’s group systematically characterised the physicochemical properties of palladium nanoparticles as a function of size over non-reducible supports to quantify structure–function relations in allylic alcohol selox [133–137, 158, 159]. The combination of XPS and XAS measurements revealed that freshly prepared alumina [134, 137] and silica [135, 158] supported nanoparticles are prone to oxidation as their diameter falls below ~4 nm, with the fraction of PdO proportional to the support surface area and interconnectivity. Complementary kinetic analyses uncovered a direct correlation between the surface PdO content and activity/TOFs towards cinnamyl and crotyl alcohol selox [134, 137]. Operando liquid phase XAS of Pd/C and Pd/Al_2_O_3_-SBA-15 catalysts during cinnamyl alcohol selox evidenced in situ reduction of PdO (Fig. 13), however, by virtue of simultaneously measuring the rate of alcohol selox, Lee et al. were able to prove that this oxide → metal structural transition was accompanied by coincident deactivation. Together these findings strongly implicate a (surface) PdO active phase, consistent with surface science predictions that metallic palladium favours aldehyde decarbonylation and consequent self-poisoning by CO and organic residues [143, 160], akin to that reported during fatty acid decarboxylation over Pd/MCF [161].

**Fig. 14:**
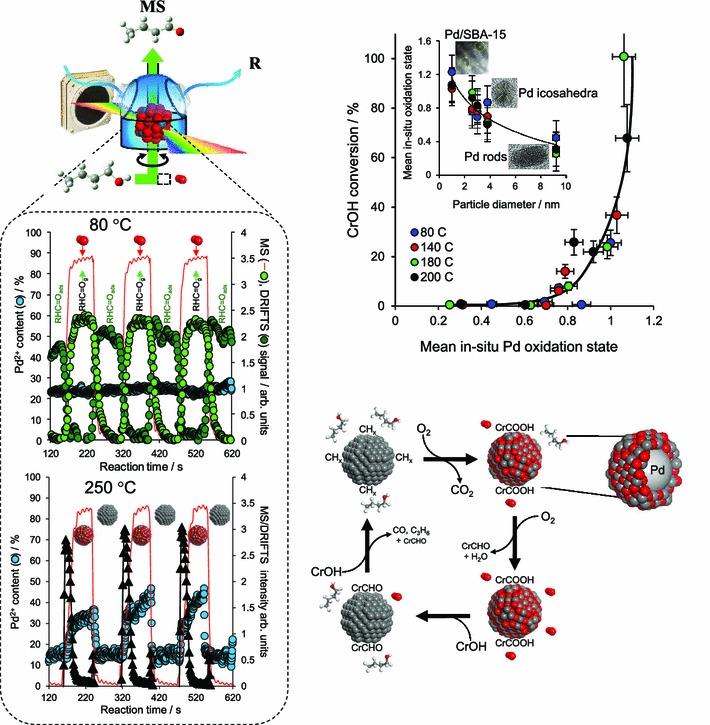
(*Left*) Cartoon of operando DRIFTS/MS/XAS reaction cell and resulting temperature dependent behaviour of Pd oxidation state and associated reactivity towards crotyl alcohol oxidation over a Pd/meso-Al_2_O_3_ catalyst—only selective oxidation over surface PdO occurs at 80 °C, whereas crotonaldehyde decarbonylation and combustion dominate over Pd metal at 250 °C; (*top right*) relationship between Pd oxidation derived in situ and crotyl alcohol conversion; (*bottom right*) summary of reaction-induced redox processes in Pd-catalysed crotyl alcohol selox. Adapted with permission from references [166, 168]. Copyright 2011 and 2012 American Chemical Society

To conclusively establish whether oxide or metal is responsible for alcohol selox catalysed by dispersed palladium nanoparticles, a multi-dimensional spectroscopic investigation of vapour phase crotyl alcohol selox was undertaken (since XAS is an averaging technique a complete understanding of catalyst operation requires multiple analytical techniques [162–164]). Synchronous, time-resolved DRIFTS/MS/XAS measurements of supported and colloidal palladium were performed in a bespoke environmental cell [165] to simultaneously interrogate adsorbates on the catalyst surface, Pd oxidation state and reactivity under transient conditions in the absence of competitive solvent effects [166, 167]. Under mild reaction temperatures, palladium nanoparticles were partially oxidised, and unperturbed by exposure to sequential alcohol or oxygen pulses (Fig. 14). Crotonaldehyde formed immediately upon contact of crotyl alcohol with the oxide surface, but only desorbed upon oxygen co-adsorption. Higher reaction temperatures induced PdO reduction in response to crotyl alcohol exposure, mirroring that observed during liquid phase selox, however, this reduction could be fully reversed by subsequent oxygen exposure. Such reactant-induced restructuring was exhibited by all palladium nanoparticles, but the magnitude was inversely proportional to particle size [168]. These dynamic measurements decoupled the relative reactivity of palladium oxide from metal revealing that PdO favoured crotyl alcohol selox to crotonaldehyde and crotonic acid, whereas metallic palladium drove secondary decarbonylation to propene and CO in accordance with surface science predictions [143].

**Fig. 15:**
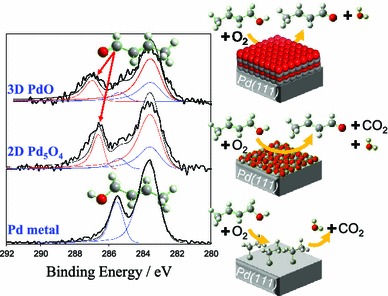
(*Left*) C 1s XP spectra of crotyl alcohol/O_2_ gas mixture over metallic and oxidised Pd(111) surfaces; (*right*) differing reactivity of palladium metal and oxide surfaces. Adapted from reference [169]. Copyright 2012 American Chemical Society

Recent ambient pressure XPS investigations of crotyl alcohol/O_2_ gas mixtures over metallic and oxidised Pd(111) single crystal surfaces confirmed that only two-dimensional Pd_5_O_4_ and three-dimensional PdOx surfaces were capable of crotonaldehyde production (Fig. 15) [169]. However, even under oxygen-rich conditions, on-stream reduction of the Pd_5_O_4_ monolayer oxide occurred >70 °C accompanied by surface poisoning by hydrocarbon residues. In contrast, PdOx multilayers were capable of sustained catalytic turnover of crotyl alcohol to crotonaldehyde, conclusively proving surface palladium oxide as the active phase in allylic alcohol selox.

### Establishing support effects

**Fig. 16:**
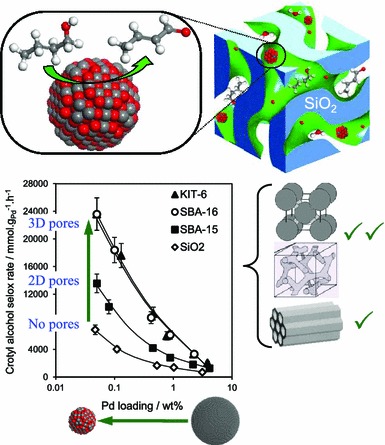
Comparative activity of Pd nanoparticles dispersed over amorphous, 2D non-interconnected SBA-15 and 3D interconnected SBA-16 and KIT-6 mesoporous silicas in the selective aerobic oxidation of crotyl alcohol. Adapted from reference [135]. Copyright 2011 American Chemical Society

Anchoring Pd nanoparticles onto support structures offers an effective means to tune their physicochemical characteristics and prevent on-stream deactivation e.g. by sintering. Supports employing porous architectures, acid/base character and/or surface redox chemistry e.g. strong metal support interaction (SMSI), afford further opportunities to influence catalyst reactivity [170–173]. Mesoporous silicas are widely used to disperse metal nanoparticles [135, 136, 171, 174, 175]. The transition from low surface area, amorphous silica (200 m^2^g^−1^) to two-dimensional non-interconnected pore channels (SBA-15) [73] and three-dimensional interconnected porous frameworks (SBA-16, KIT-6) [73, 176, 177] improved the dispersion of Pd nanoparticles and hence degree of surface oxidation and thus activity in allylic alcohol selox (Fig. 16), but had little impact on the mass transport of small alcohols to/from the active site. [135, 136] The high thermal and chemical stability of such mesoporous silica [178, 179] makes such supports well-suited to commercialisation. Pd nanoparticles confined within such mesoporous silicas demonstrate good selectivity in crotyl and cinnamyl alcohol selox to their respective aldehydes (>70 %), and excellent TOFs of 7,000 and 5,000 h^−1^ for the respective alcohols. Similar activities are reported for secondary and tertiary allylic alcohols, highlighting the versatility of silica supported Pd nanoparticles [51, 135, 136, 180–182]. Incorporation of macropores into SBA-15 via dual hard/soft templating to form a hierarchically ordered macroporous–mesoporous Pd/SBA-15 was recently shown to promote the catalytic selox of sterically challenging sesquiterpenoid substrates such as farnesol and phytol via (1) stabilising PdO nanoparticles and (2) dramatically improving in-pore diffusion and access to active sites [158].

**Fig. 17:**
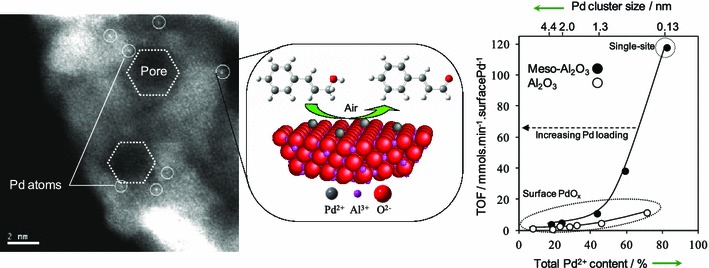
(*Left*) HAADF–STEM image of atomically dispersed Pd atoms on a mesoporous Al_2_O_3_ support; and (*right*) associated relationship between Pd^2+^ content/dispersion and activity in crotyl alcohol selox over Pd/alumina catalysts. Adapted with permission from reference [137]. Copyright Wiley–VCH Verlag GmbH & Co. KGaA

The benefits of mesostructured supports are not limited to silica, with ultra-low loadings of palladium impregnated onto a surfactant-templated mesoporous alumina (350 m^2^ g^−1^) generating atomically dispersed Pd^2+^ centres [137]. Such single-site catalysts were 10 times more active in crotonaldehyde and cinnamaldehyde production than comparable materials employing conventional (100 m^2^ g^−1^) γ-alumina, owing to the preferential genesis of higher concentrations of electron-deficient palladium [134, 137], due to either pinning at cation vacancies or metal → support charge transfer [183]. These Pd/meso-Al_2_O_3_ catalysts exhibited similar TOFs to their silica counterparts (7,080 and 4,400 h^−1^ for crotyl and cinnamyl alcohol selox, respectively) [137], consistent with a common active site and reaction mechanism (Fig. 17).

Mesoporous titania and ceria have also attracted interest as novel catalyst supports. The oxygen storage capacity of ceria-derived materials is of particular interest due to their facile Ce^3+^↔Ce^4+^ redox chemistry [173, 184–188]. Sacrificial reduction of the ceria supports by reactively formed hydrogen liberated during the oxidative dehydrogenation of alcohols could mitigate in situ reduction of oxidised palladium, and hence maintain selox activity and catalyst lifetime, with Ce^4+^ sites regenerated by dissociatively adsorbed gas phase oxygen [187, 189, 190]. Due to its high density, conventional nanocrystalline cerias possess meagre surface areas (typically ~5 m^2^g^−1^), hence Pd/CeO_2_ typically exhibit poor selox behaviour due to their resultant low nanoparticle dispersions which favour (self-poisoning) metallic Pd [189, 191, 192].

### Bimetallic palladium selox catalysts

Incorporation of a second metal into palladium catalysts can improve both alcohol selox stability and selectivity. Typical promoters such as Ag, Bi, Pb and Sn [157, 193–196], enhance oxidation performance towards challenging substrates such as propylene glycol [197] as well as allylic and benzylic alcohols. Wenkin et al. [194] reported glucose oxidation to gluconates was increased by a factor of 20 over Pd–Bi/C catalysts (Bi/Pd_s_ = 0.1) versus Pd/C counterparts. In situ XAS and attenuated total reflection infrared spectroscopy (ATR-IR) suggested that Bi residing at the catalyst surface protects palladium from deactivation by either over-oxidation (a hypothesis since disproved [166, 167, 169]) or site-blocking by aromatic solvents [153]. Prati et al. [200] first reported significant rate enhancements and resistance to deactivation phenomena in the liquid phase selox of d-sorbitol to gluconic/gulonic acids upon addition of Au to Pd/C and Pt/C materials [198], subsequently extended to polyol and long chain aliphatic alcohols [199]. A strong synergy between Pd and Au centres was also demonstrated by Hutchings et al., wherein Au–Pd alloy nanoparticles supported on titania exhibited increased reactivity towards a diverse range of primary, allylic and benzylic alkyl alcohols compared to monometallic palladium analogues. The versatility of Au–Pd catalysts has also been shown in selox of saturated hydrocarbons [201], ethylene glycol [202], glycerol [203] and methanol [204], wherein high selectivity and resistance to on-stream deactivation is noted.

**Fig. 18:**
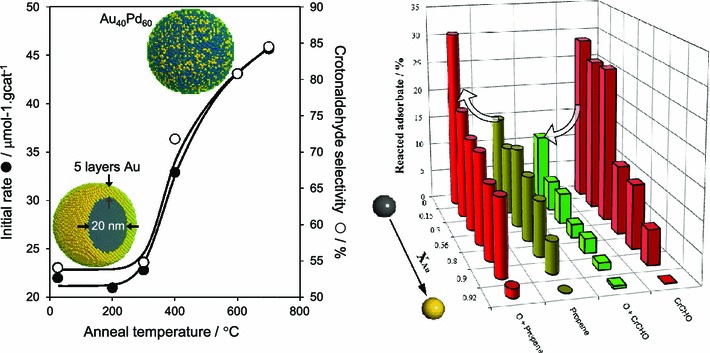
Impact of thermally induced Au–Pd alloying of (*left*) titania-supported Au shell–Pd core nanoparticles on crotyl alcohol aerobic selox adapted from reference [208], with permission from Elsevier; and (*right*) ultrathin gold overlayers on Pd(111) on crotonaldehyde and propene decomposition with/without co-adsorbed oxygen, adapted from reference [160] with permission from the PCCP Owner Societies

The effect of Au–Pd composition has been extensively studied for bimetallic nanoparticles stabilised by PVP surfactants [205]. An optimal Au:Pd composition of 1:3 was identified for 3 nm particles towards the aqueous phase aerobic selox of benzyl alcohol, 1-butanol, 2-butanol, 2-buten-1-ol and 1,4-butanediol; in each case the bimetallic catalysts were superior to palladium alone. Mertens et al. [206] examined similar systems utilising 1.9 nm nanoparticles, wherein an optimal Au content of around 80 % was determined for benzyl alcohol selox. The synergic interaction between Au and Pd therefore appears interdependent on nanoparticle size. It is well-known that the catalytic activity of Au nanoparticles increases dramatically <2 nm [207], hence it is interesting to systematically compare phase separated and alloyed catalysts. The author’s group prepared titania-supported Au shell (5-layer)-Pd core (20 nm) bimetallic nanoparticles for the liquid phase selox of crotyl alcohol and systematically studied the evolution of their bulk and surface properties as a function of thermal processing by in situ XPS, DRIFTS, EXAFS, XRD and ex-situ HRTEM. Limited Au/Pd alloying occurred below 300 °C in the absence of particle sintering [208]. Higher temperatures induced bulk and surface alloying, with concomitant sintering and surface roughening. Migration of Pd atoms from the core to the surface dramatically enhanced activity and selectivity, with the most active and selective surface alloy containing 40 atom % Au (Fig. 18). This discovery was rationalised in terms of complementary temperature-programmed mass spectometric studies of crotyl alcohol and reactively formed intermediates over Au/Pd(111) model single crystal catalysts which reveal that gold–palladium alloys promote desorption of the desired crotonaldehyde selox product while co-adsorbed oxygen adatoms actually suppress aldehyde combustion. In contrast, the combustion of propene, the undesired secondary product of crotonaldehyde decarbonylation, is enhanced by co-adsorbed oxygen [160].

Scott et al. prepared the inverse Au core-Pd shell nanoparticles and explored the catalytic cycle for alcohol selox to assess their associated stability [205, 209–212]. In situ Pd–K and Pd–L_III_ edge XAS of a Au nanoparticle/Pd(II) salt solution were undertaken to discriminate two possible reaction mechanisms. No evidence was found that crotyl alcohol oxidation was accompanied by Pd^2+^ reduction onto Au nanoparticles, resulting in the formation of a metallic Pd shell (with oxygen subsequently regenerating electron-deficient palladium), and therefore proposed β-H elimination as the favoured pathway. Scott and co-workers proposed that the Au core prevents the re-oxidation of surface Pd^0^ atoms; no Pd–O and Pd–Cl contributions were observed by EXAFS.

In summary, the selective oxidation of complex alcohol substrates can be accomplished through Pd-mediated heterogeneous catalysis with high turnover and product selectivity. Application of in situ and operando techniques, such as X-ray and IR spectroscopies, has elucidated the mechanism of alcohol oxidative dehydrogenation and competing aldehyde decarbonylation. Surface PdO has been identified as the active catalytic species, and deactivation the result of reduction to metallic palladium and concomitant self-poisoning by strongly bound CO and carbonaceous residues. Breakthroughs in analytical tools and synthetic approaches to engineering nanoporous supports and shape/size controlled nanoparticles have delivered significant progress towards improved atom and energy efficiency and catalyst stability, however, next generation palladium selox catalysts necessitate improved synthetic protocols to create higher densities of ultra-dispersed Pd^2+^ centres with superior resistance to on-stream reduction under atmospheric oxygen.
